# Blue Nevus of the Hard Palate: The Importance of a Careful Examination in an Emergency Setting

**DOI:** 10.1155/2022/6329334

**Published:** 2022-02-15

**Authors:** Ana Teresa Tavares, André Pereira, João Pimentel, Marcelo Prates, Luís Fonseca, Maria Rosário Marques, Francisco Proença

**Affiliations:** ^1^Stomatology Department, Centro Hospitalar Universitário de Lisboa Central, Lisbon, Portugal; ^2^Pathology Department, Centro Hospitalar Universitário de Lisboa Central, Lisbon, Portugal

## Abstract

Oral common blue nevus is an asymptomatic, benign, rare, pigmented lesion and sometimes clinically indistinguishable from other pigmented lesions such as the cellular blue nevus or early-stage malignant melanoma. Since it shows clinical similarities with a malignant lesion and with cellular blue nevus that can itself suffer malignant transformation, the decisive diagnosis is crucial for adequate treatment, follow-up, and prognosis. Diagnosis confirmation is given by histological analysis, the reason why most oral pigmented lesions are excised. The following case presents an asymptomatic oral pigmented lesion of the hard palate discovered during observation in an emergency setting due to an abscess of dental origin. The lesion was fully excised, and histological examination reported a “common blue nevus.” In this case, we intend to present a rare lesion of the oral cavity and the importance of performing a routine oral examination when given a chance as a preventive approach.

## 1. Introduction

Oral melanocytic nevi (OMN) are rare, benign tumors of melanocytes [1–7]. The pathogenesis and etiology of OMN are still poorly understood [1, 2, 4, 5, 8, 9]; the most consensual theory is that they arise from latent dendritic melanocytes, which remain trapped in the dermis, from the embryologic migration of melanoblasts, their precursors, and from the neural crest to the epidermis [1, 3, 4, 10–12]. OMN can be congenital or acquired with higher prevalence of the latter [4, 5, 9, 13] and are histologically classified as junctional, compound, intradermic (intramucosal), combined, and blue [1–3, 7]. This classification depends on distribution, localization, and morphology of the nevus cells [1–3]. The most common OMN is the intramucosal nevus followed by the blue nevus (19–36%) [1–7, 14]. Blue nevus itself has several histological classifications from which can be clinically indistinct: common blue nevus, cellular blue nevus (CBN), and atypical nevus [1, 3, 5, 7, 9, 10, 13, 15].

**Figure 1:**
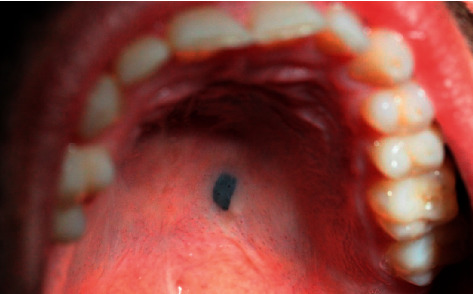
Palatal lesion during oral examination.

**Figure 2:**
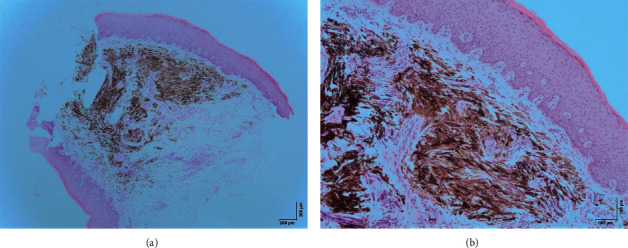
Microscopic examination at magnifications 40x (a) and 100x (b) with hematoxylin and eosin. There is a noncircumscribed submucosal proliferation of elongated spindle-shaped melanocytes that are grouped in short fascicles some of them arranged parallel to the overlying epithelium. The melanocytes contain a variable amount of melanin pigments in the cytoplasm. Some fibrosis is observed in between the fascicles of pigmented cells. There is no atypia or areas of necrosis. No mitoses were observed.

**Figure 3:**
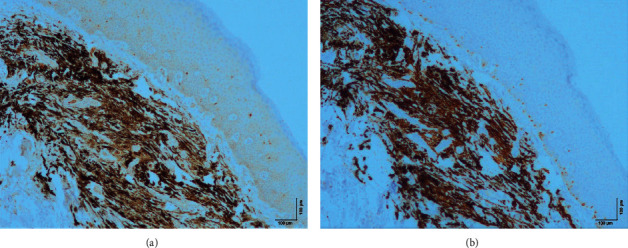
Microscopic examination at magnification 100x. Nevus cells stain for MelanA (a) and S100 (b).

Oral blue nevus has a prevalence of 0.1% in the general population, appears mainly from the third to the fifth decades, and women tend to be more affected than men [2, 4, 7, 14]. It develops more frequently in the hard palate (69%), followed by the labial mucosa and vermillion border [1–3, 5–7, 14]. Most lesions are asymptomatic, and therefore, its oral presentation is mostly discovered during routine dental examinations [2, 15]. Clinically, the common blue nevus usually presents as a solitary, smooth-surfaced, well-defined, flat, or elevated papule with a diameter of less than 10 mm, though wider lesions have been described [1, 2, 5, 7, 9, 11, 13]. Its color varies from brown to blue, depending on the pigment quantity and depth or location [2–5, 13]. Histologically, the diagnostic cell of the blue nevus is a pigmented, spindle-shaped dendritic melanocyte with a slender, branching network of dendritic processes without atypia or mitotic figures [1–3, 7, 10–13]. The nuclei are small, elongated, and hyperchromatic [2, 11, 13]. Blue nevus' cells are frequently positive to S100, MelanA (MART-1), and HMB-45 [2, 4, 11, 13]. Literature states that the risk of malignant transformation of CBN into oral malignant melanoma (OMM) is estimated to be between 5.2% and 6.3% [9]. Because of multiple reports of tumors with features combined between CBN and OMM, the controversial designation of “atypical blue nevus” or “atypical cellular blue nevus” was created for these borderline lesions [8, 10, 14, 15]. However, despite the efforts, there is still no uniform histological criteria to differentiate atypical CBN from OMM [14, 16].

The following case presents a 40-year-old male with an asymptomatic pigmented lesion located in the left hard palate, discovered during an emergency visit due to a dental abscess. The lesion was fully excised with the histological diagnosis of “common blue nevus of the oral mucosa.” This case report presents a rare lesion of the oral cavity that has a risk of malignant transformation, thus highlighting the importance of the routine oral examination as a preventive approach.

## 2. Case Presentation

A 40-year-old male presented in the Stomatology Emergency Room with chief complaint of toothache in the fourth quadrant. He had a history of treated pulmonary tuberculosis, high blood pressure, abdominal surgery after trauma, and obstructive sleep apnea-hypopnea syndrome, treated with continuous positive airway pressure (CPAP). He was medicated with nebivolol, valproic acid, bromazepam, and risperidone and slept with continuous positive airway pressure. He denied any history of alcohol intake or smoking habits. Regarding family history, he alleged to have had an aunt with oral cancer.

While being diagnosed with an acute apical periodontitis of the inferior right first molar, during oral examination, it was found a black melanocytic-type lesion on the posterior half of the left hard palate (Figure 1). The lesion measured 5 mm × 2.5 mm, was flat, homogeneous, well-defined, nonulcerated, and painless. The patient had no recollection of its existence. No masses or nodes were palpable in the cervical or preauricular areas.

The acute dental infection was primarily addressed. Regarding palatal lesion, given the fact that he was unaware of the palatal lesion, and therefore there was no way of knowing if it had a recent growth and due to his alleged family history, an excisional biopsy was scheduled and blood analysis was requested.

The blood tests revealed normal, besides slightly elevated hemoglobin (17.9 × 10 g/L).

The biopsy was performed under local anesthesia with lidocaine + adrenaline (1 : 80000) and a number 15 scalpel blade. The lesion was excised with a 2 mm margin, and local hemostasis was obtained with a periodontal dressing.

Histological examination of the lesion revealed a “common blue nevus of oral mucosa” (Figures 2 and 3).

**Figure 4:**
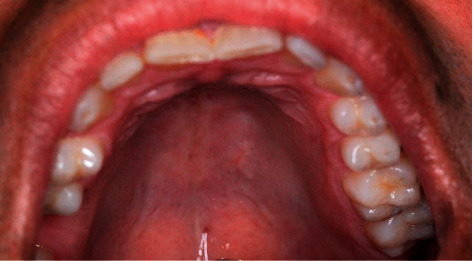
Surgical wound 4 months after biopsy.

Upon follow-up, the patient remains asymptomatic, and the surgical wound is fully closed (Figure 4), with no signs of clinical recurrence.

## 3. Discussion

Blue nevus is very rarely seen in the oral cavity [2, 3, 7, 14]. Since they are usually asymptomatic, their finding in a routine oral examination is frequent [2, 5].

Although the ABCDE system may be a helpful tool in considering malignancy in the oral cavity, clinical impression is insufficient for definite diagnosis and most pigmented lesions end up excised [2, 17]. This procedure should also be considered in these local findings: pain, swelling, recent growth, color changes, bleeding, and ulceration [2].

Due to the fact that most oral pigmented lesions are benign, excision usually is required only when the patient reports discomfort [2]; however, performing an incisional biopsy of oral pigmented lesions with malignant potential is an area of controversy among researchers [2, 3]. Some authors defend that cutting into malignant lesions when performing an incisional biopsy can result in increased risk of local recurrence or regional or distant metastasis, but several studies have also refuted this hypothesis [2, 3, 17, 18]. The extent of the surgical margins when excising oral pigmented lesions is still in debate [2]. Australia and New Zealand guidelines for cutaneous melanoma recommend an initial excisional biopsy with 2 mm margins whenever possible [2, 19], but no OMM parallel has yet been established.

Around 30% of all OMM develop from previous existing pigmented lesion, while most are *de novo* [20]. Still, primary OMM is an infrequent but very aggressive lesion, accounting for 0.5% of all oral malignancies [2, 4, 6, 13, 14, 17] and less than 1% of all melanomas [13, 19, 20]. Its 5-year survival rate ranges from 10–45% [7]. Malignant transformation or recurrence of a blue nevus, though extremely rare, can in fact develop [2, 7, 16, 21]; most often, if not always, its stated that it occurs in cellular types, although this is still subject to debate [2, 3, 6, 7, 11, 15].

Some oncogenic protein mutations have been described in common nevi and in both cutaneous and oral melanoma; the most consensual finding is a mutation in Bioinformatics Resources and Applications Facility (BRAF). The suggested mechanism for this association is that BRAF mutations trigger the proliferation of melanocytic cells, thus forming a nevus. When a growth-arrest response occurs, the proliferation ceases. This mutation, however, is only found in less than 10% of OMM compared to 80% found in cutaneous melanoma; furthermore, some studies suggest that blue nevus rarely has such mutation, which may imply that they represent a distinct type of melanocytic neoplasms [1, 5, 11, 22].

The final diagnosis is therefore of utmost relevance since treatment and prognosis of benign pigmented lesions are far different from OMM [3, 8].

This case presents the uncertainty of malignancy of an oral pigmented lesion which is, by itself, very rare, and data are scarce regarding its proper approach. An oral examination in an emergency setting regarding dental problems revealed the lesion. An excision was performed, with the positive reassurance that only an excisional biopsy can provide, that no malignancy was found associated with the palatal lesion.

## Data Availability

No data were used to support this study.

## References

[1] Ferreira L., Jham B., Assi R., Readinger A., Kessler H. P. (2015). Oral melanocytic nevi: a clinicopathologic study of 100 cases. *Oral Surgery, Oral Medicine, Oral Pathology and Oral Radiology*.

[2] Toeda Y., Uzawa K., Yamano Y. (2016). Blue nevus of the hard palate: a case report. *Journal of Oral and Maxillofacial Surgery, Medicine, and Pathology*.

[3] Santos T. d. S., Frota R., Martins-Filho P. R. S., Cavalcante J. R., Raimundo R. d. C., Andrade E. S. d. S. (2011). Extenso nevo azul intraoral: relato de caso. *Anais Brasileiros de Dermatologia*.

[4] Meleti M., Vescovi P., Mooi W. J., van der Waal I. (2008). Pigmented lesions of the oral mucosa and perioral tissues: a flow-chart for the diagnosis and some recommendations for the management. *Oral Surgery, Oral Medicine, Oral Pathology, Oral Radiology & Endodontics*.

[5] Meleti M., Mooi W. J., Casparie M. K., van der Waal I. (2007). Melanocytic nevi of the oral mucosa–No evidence of increased risk for oral malignant melanoma: an analysis of 119 cases. *Oral Oncology*.

[6] Buchner A., Merrell P. W., Carpenter W. M. (2004). Relative frequency of solitary melanocytic lesions of the oral mucosa. *Journal of Oral Pathology and Medicine*.

[7] Pinto A., Raghavendra S., Lee R., DeRossi S., Alawi F. (2003). Epithelioid blue nevus of the oral mucosa: a rare histologic variant. *Oral Surgery, Oral Medicine, Oral Pathology, Oral Radiology & Endodontics*.

[8] Hasney C., Butcher R. B., Amedee R. G. (2008). Malignant melanoma of the head and neck: a brief review of pathophysiology, current staging, and management. *The Ochsner Journal*.

[9] Kesty K., Zargari O. (2015). Eruptive blue nevi. *Indian Journal of Dermatology, Venereology and Leprology*.

[10] Scheller K., Scheller C., Becker S., Holzhausen H.-J., Schubert J. (2010). Cellular blue nevus (CBN) lymph node metastases of the neck with no primary skin lesion: a case report and review of literature. *Journal of Cranio-Maxillofacial Surgery*.

[11] Zembowicz A., Phadke P. A. (2011). Blue nevi and variants: an update. *Archives of Pathology & Laboratory Medicine*.

[12] Moshaver A., Puttagunta L., Seikaly H. (2006). Malignant blue nevus of the parotid gland: a case report. *Head & Neck*.

[13] Zembowicz A., Mihm M. C. (2004). Dermal dendritic melanocytic proliferations: an update. *Histopathology*.

[14] Hicks M. J., Flaitz C. M. (2000). Oral mucosal melanoma: epidemiology and pathobiology. *Oral Oncology*.

[15] Barnhill R. L., Argenyi Z., Berwick M. (2008). Atypical cellular blue nevi (cellular blue nevi with atypical features): lack of consensus for diagnosis and distinction from cellular blue nevi and malignant melanoma (“malignant blue nevus”). *The American Journal of Surgical Pathology*.

[16] Shumway B. S., Rawal Y. B., Allen C. M., Kalmar J. R., Magro C. M. (2013). Oral atypical cellular blue nevus: an infiltrative melanocytic proliferation. *Head and Neck Pathology*.

[17] Meleti M., Leemans C. R., Mooi W. J., Vescovi P., van der Waal I. (2007). Oral malignant melanoma: a review of the literature. *Oral Oncology*.

[18] Bong J. L., Herd R. M., Hunter J. A. A. (2002). Incisional biopsy and melanoma prognosis. *Journal of the American Academy of Dermatology*.

[19] Guidelines C. P., Zealand N. (2008). *Management Melanoma*.

[20] Castillo S. A., Pham A. K., Lefferts J. A., Yan S., Bridge J. (2018). A diagnostically-challenging case of melanoma ex blue nevus with comprehensive molecular analysis, including the 23-gene expression signature (myPath melanoma). *Journal of Cutaneous Pathology*.

[21] Andrei R., Zurac S., Socoliuc C., Mandisodza P., Staniceanu F. (2015). Problems of differential diagnosis in melanoma arising from blue naevus. *Romanian Journal of Internal Medicine = Revue Roumaine de Medecine Interne*.

[22] Zito P. M. (2019). Cancer. *Oral Melanoma Pathophysiology Histopathology Treatment/Management*.

